# Group Selection has the Potential to Increase Yield and Resource Use Efficiency in Cereal Crops

**DOI:** 10.1111/eva.70347

**Published:** 2026-11-11

**Authors:** Susanna Vain, Marina Semchenko, Ülle Tamm, Ilmar Tamm, John A. Davison, Kristjan Zobel

**Affiliations:** ^1^ Institute of Ecology and Earth Sciences University of Tartu Tartu Estonia; ^2^ Department of Research Centre of Estonian Rural Research and Knowledge Jõgeva Estonia

**Keywords:** crop ecology, crop roots, evolutionary agroecology, group selection, plant breeding

## Abstract

The initial stages of crop breeding rely on selecting the best performing individuals, despite these often possessing competitive traits that hinder collective yield. Group selection, where the best‐performing groups, rather than individuals, form subsequent generations, is one proposed solution but lacks empirical support. Here we present the results of a group selection experiment targeting protein yield in oat and barley. After eight generations, group‐selected oat and barley plants produced higher protein and grain yield than the first generation of plants in the target environment at the beginning of the experiment. Although yield showed an overall positive linear trend across generations, the negative quadratic component indicated a deceleration of this increase, suggesting that further gains may not continue at the same rate. When yield trends were compared with concurrently grown registered cultivars, group‐selected plants had higher grain as well as protein yield in barley, whereas no similar trend was detected in oat. Selection for higher group‐level yield resulted in a gradual decrease in root production, increased straw production, and sturdier aboveground structures. These trait shifts suggest that increased yield may have been achieved through more efficient allocation to resource‐capturing organs. Although the present experimental design does not allow us to fully disentangle the effects of group selection from other processes, such as genetic drift or heterosis, our results indicate that group selection may nevertheless hold potential for improving crop yield and addressing key challenges in sustainable agriculture, warranting further empirical investigation.

## 1 Introduction

Group selection may not be a credible evolutionary force in natural systems (Leigh Jr 2010), but it could be applicable for achieving the aims of artificial selection. Indeed, it has been suggested that crop breeding should be based on group selection to overcome the mismatch between strategies that confer individual and collective fecundity (Denison et al. 2003; Montazeaud et al. 2020; Weiner et al. 2010; Zhu et al. 2018; Murphy et al. 2017). Densely growing plants always compete for light, water and nutrients. Thus, individual‐level selection identifies plants that are best at acquiring resources in a competitive environment (Anten and Vermeulen 2016; Gersani et al. 2001). Superior competitive ability is achieved by investing more biomass into resource‐acquiring organs, such as leaves, stems and roots, but this investment comes at a price—fewer resources remain to invest into seed production (Anten and Vermeulen 2016). Such pattern has also been documented in annual cereal crops, where several empirical studies have demonstrated positive relationship between competitive ability and investment in resource‐acquiring organs (Zhu et al. 2018; Zhu et al. 2022; Zhu and Zhang 2013). This evolutionary outcome seems to be inevitable in most natural settings due to the so‐called prisoner's dilemma (Axelrod and Hamilton 1981) – collective seed production would be higher if all individuals limited their production of resource‐acquiring organs to the optimum level; yet genotypes that overinvest into competitive organs outcompete neighbors that invest at the optimum level by taking more than equal share of resources (Anten and Vermeulen 2016; Rankin et al. 2007). Hence, the evolutionary arms race for superior resource acquisition often results in inefficient resource use in plant communities (i.e., a low efficiency of converting acquired resources into seeds) because the target of natural selection is typically individual performance rather than the seed production of the whole population. By contrast, the target of crop breeding is precisely the latter.

Natural selection shaped the morphology and growth of crop wild relatives long before their domestication, and its legacy continues to operate in modern crop varieties (Goldberg 1990; Milla et al. 2014; Milla et al. 2015). Discussions about less competitive communal non‐rice cereal crop plants have circulated ever since Donald introduced the communal wheat ideotype (Donald 1968). He stated that crop plants should be shorter and have more upright leaves and fewer tillers in order to avoid competition for space and light with neighboring plants. Breeding for weak competitor crops received a lukewarm reception from plant breeders, since weak competitors have generally been associated with weak plants (Reynolds et al. 1994; Zhang et al. 1999). Nevertheless, the competitive ability of crop plants has notably decreased during recent decades. The most remarkable example of this was the emergence of short but sturdy varieties during the Green Revolution in the 1960s (Donald 1981). The main motivation for breeding shorter varieties was to increase resistance to lodging under increased fertilizer load. However, because plant height is a crucial trait conferring superiority in competition for light, breeding coincidentally resulted in decreased competitive ability for light. Likewise, root system volume has decreased with breeding despite the fact that roots have never been the direct target of cereal breeding (Zhu et al. 2022; Waines and Ehdaie 2007; Zhai et al. 2016; Aziz et al. 2017; Fradgley et al. 2020; Zhu et al. 2019). Hence, despite not being consciously selected for, the competitive ability of crop plants has decreased over time, but there probably remains ample room for improvement if allocation to competitive traits is treated as a direct target of crop breeding.

In the early stages of conventional cereal breeding, the individual plants with highest yield, among other features, such as disease resistance or dry matter partitioning (e.g., harvest index) are selected (Figure 1A). Typically, candidate plants are first grown and selected on an individual basis, then grown in groups where also individual plants are selected (Yan 2021; Acquaah 2012). Only in later stages plants are grown in groups and whole‐plot yield is taken into account as one of the primary deciding factors for selection. However, this selection method ignores phenotypic variation in plant competitive behavior when selecting among plants grown in the absence of competition, and inadvertently favors individuals with high investment into competitive organs when selection takes place in dense plant stands. Therefore, the potential to increase yield by restraining plant competitive behavior is not realized. Group selection is a possible approach for maximizing collective grain yield. In group selection, instead of selecting the best performing individuals, the best performing groups are selected and provide the basis of the following generation (Zhu et al. 2019; Figure 1B). In the presence of sufficient genetic variation along the trade‐off between seed yield and investment into competitive behavior, group selection should reduce suboptimal investment into competitive organs and enhance traits that allow crop plants to maintain high seed production, even when grown in dense stands. The best empirical evidence for successful group selection and inadvertent selection for reduced competitive behavior comes from animal breeding. In an experiment with hens, Muir (Muir 1996) selected for the best group‐level egg production, which coincidentally also led to reduced aggressiveness in hens (a competitive trait). Group selection can also be successfully applied to plants (Goodnight 1985), and a major effect locus associated with increased group‐level productivity in high‐density stands has been identified in *Arabidopsis* (Wuest et al. 2022; Wuest and Niklaus 2018). However, the approach has not yet been applied to crop breeding.

**FIGURE 1:**
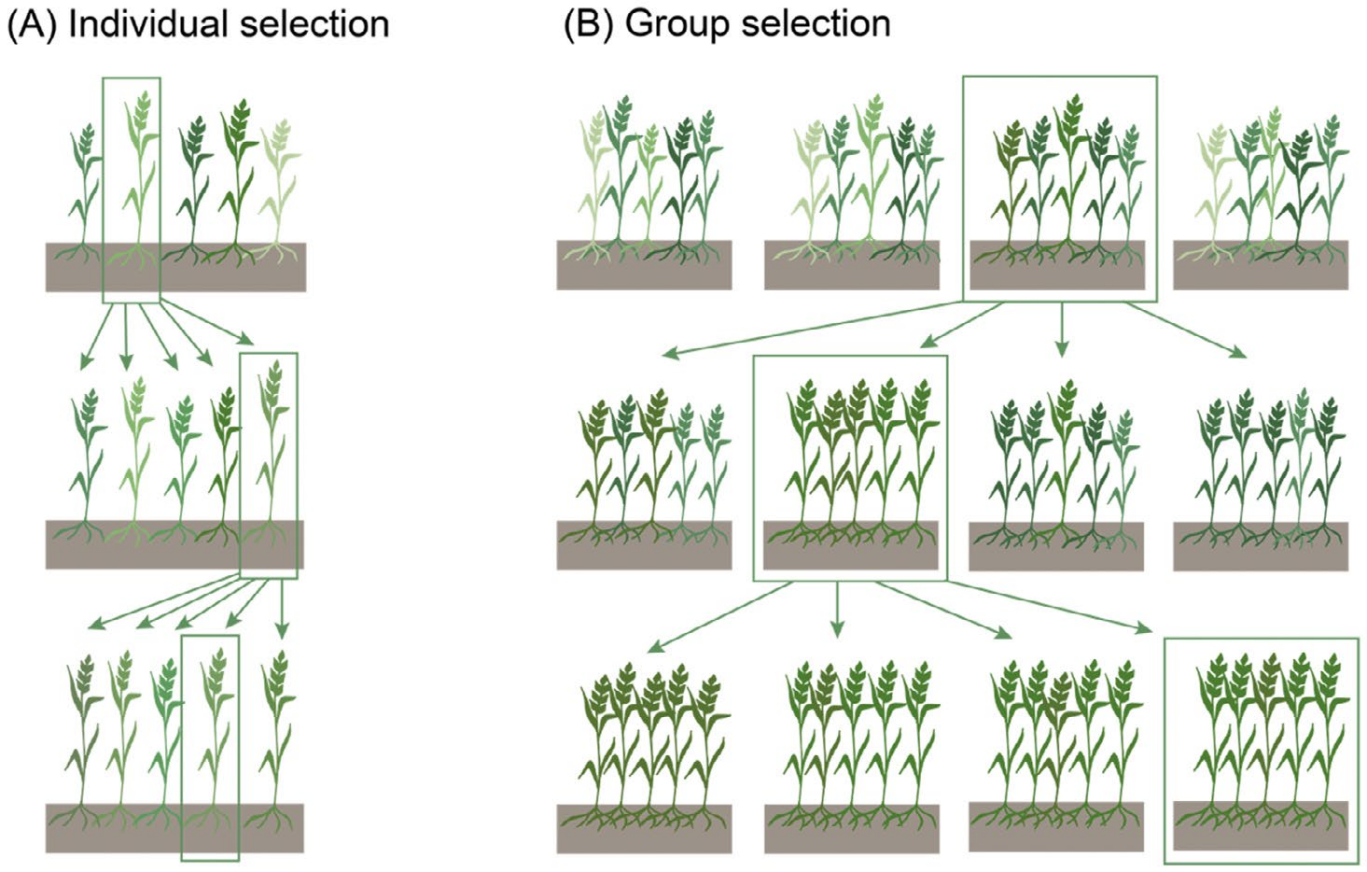
Principles of individual and group selection in plant breeding. (A) Individually superior plants are selected under individual selection, which is typically applied in the initial stages of conventional plant breeding. Individual superiority is achieved by competitively acquiring a larger share of resources at the expense of others. Hence, highly competitive genotypes are favored under individual selection; when grown in monocultures during later stages of breeding, the full yield potential is not realized due to excessive resources being directed to production of competitive organs. (B) Under group selection, plants are grown in dense stands and groups, rather than individuals, with the highest‐yield group giving rise to the following generation. Group selection helps to identify genotypes that avoid excessive allocation to competitive behavior and can maintain high seed production when grown in dense groups. Group‐selected plants are competitively inferior to individually selected plants but have higher collective yield when grown in dense stands, which is the desired agronomic outcome.

Here we present the results of a group selection experiment using cereal crops. We applied group selection to original crosses of oat and barley for eight generations. Given that protein yield is a key factor determining the nutritional quality of the harvest, we chose protein yield as the trait under selection. Thus, each generation was sown from seeds originating from the group with the highest collective protein yield. To speed up the selection process, we grew two generations per year – a winter generation in a greenhouse and a summer generation in the field. Since the greenhouse generations experienced artificial growing conditions, and crop field performance is most informative for agronomic applications, we focus on summer generations to assess the success of the selection process.

## 2 Materials and Methods

### 2.1 Experiment With Oat and Barley

In 2015, 18 original crosses of oat ( *Avena sativa* L.) and 20 crosses of barley ( *Hordeum vulgare* L.) were constructed for a group selection experiment by the Plant Breeding Department of the Centre of Estonian Rural Research and Knowledge. The parents of the crosses were modern cultivars adapted to the conditions of the region. For the next 2 years, the crosses were grown, without any selection pressure, for seed multiplication purposes. By the F3 generation, enough seed had been produced to sow crosses in the field, onto 2 × 1 m plots at a density of 700 germinating seeds per m^2^ (row spacing 11 cm), with seeds from each cross sown on its own plot (38 plots in total). This sowing density is approximately 1/3 higher than the recommended sowing density for the region (Estonian Seed Association recommended sowing densities, 2021). We purposely chose higher sowing density to increase the likelihood of interplant interactions—competitive or cooperative (Weiner et al. 2010). Furthermore, there are examples of other studies in which cooperative restraints have become apparent only at relatively high densities (Borg et al. 2018; Gao et al. 2021; Li and Xu 2018), and using higher sowing densities in group selection experiments has been advocated (Montazeaud et al. 2020). Seeds were sown in April 2017 in Jõgeva, Estonia (58° 44′ N, 26° 23′ E), where the soil type is a Luvisol. Fertilizer was applied to the experimental area at the rate of N60 P13 K25 kg ha^−1^ before sowing. Weed control was carried out with herbicides, and insecticides were used if insect control thresholds were exceeded.

Seeds were mechanically harvested in the middle of August. Grain mass (kg ha^−1^), along with seed humidity (%) and crude protein content (%), was measured for each cross. Seed quality parameters were quantified from samples of 600 g using near‐infrared transmittance technology with the Infratec grain analyzer (FOSS, Hilleroed, Denmark). Grain dry yield (kg ha^−1^) and protein yield (kg ha^−1^) were calculated using these data. For each species, the crosses with the highest protein yield were chosen as the starting points of the group selection experiment. For oats, it was cross Kusta × Nelson, and for barley, cross Odyssey × Quench.

In the group selection experiment, oats and barley were sown twice a year, in December/January (greenhouse generations) and in April/May (field generations). The experiment started in January 2018 when 10 plots of oat crosses and 10 plots of barley crosses (Kusta × Nelson and Odyssey × Quench, respectively) were sown in 2 × 1 × 0.3 m boxes in the greenhouse of the Centre of Estonian Rural Research and Knowledge at Jõgeva (58° 44′ N, 26° 23′ E) at a sowing density of 700 germinating seeds per m^2^. Peat‐based potting soil was used in the greenhouse, to which fertilizers were added at the rate of N60 P13 K25 kg ha^−1^. Plants received additional lighting for 16 h per day. Plants were harvested in March/April. Grain yield and protein yield were obtained as described above. The seeds from the oat and barley plots with the highest protein yield were chosen as the basis for the next generation. Seeds from plants of the top‐performing plot per species were pooled together and advanced to the subsequent generation throughout the experiment, meaning that all reproductive plants in that top plot contributed to the next generation. Plots within each generation were spatially distinct, although located adjacent to each other, and managed separately. However, because each new generation originated from a single selected plot of the previous generation, successive generations were genealogically linked rather than representing independent selection lines. The next generation, consisting of 10 oat and 10 barley plots, was sown in May 2018 and harvested in August 2018. This generation and all subsequent summer generations were grown in the same experimental area and under the same agronomic conditions as the original crosses described above. Specific plot locations, however, varied year‐to‐year, as per common crop rotation practice. This methodology was repeated for the next six generations (two generations per year). Sowing density, fertilizer load and artificial illumination in the greenhouse were kept constant throughout the experiment. Due to spatial constraints, the number of groups was reduced to 8 per species starting from the fifth generation (10 groups of oat and 10 groups of barley for generations 1–4, 8 groups of oat and 8 groups of barley for generations 5–8). Average temperature as well as total precipitation from sowing until maturity for the field generations can be seen in Figure S1. The long term average precipitation amount for the period between April and August (the typical growing season of spring crops) for the region is 198 mm. Therefore, the amount of rainfall in the years our experiment was carried out was less than the average in all years, except for 2020.

In addition to seed yield and quality parameters, we measured the height (cm) of 20 randomly selected plants per plot. After seeds were harvested, we collected the straw biomass (kg) from each plot. Straw biomass in this paper excludes grain mass. Additionally, we calculated plot‐level elongation index, defined as the ratio of average plant height to straw biomass. Immediately following harvest, we also took four root samples per plot (group) from inter‐plant spaces using a soil‐corer of 4 cm diameter and 26 cm in depth. The roots were carefully washed and scanned to estimate root length (cm) using WinRhizo Pro 2008a (Regent Instruments Inc., Quebec, Canada). All biomass samples were dried at 75°C for 48 h and weighed. Additionally, we calculated root:shoot ratio (root mass per unit straw biomass) as well as root length ratio (root length per unit straw biomass). Since straw biomass used in the root:shoot ratio calculation represented straw from the entire plot, root biomass from the four samples per plot was also extrapolated to represent the entire plot area, i.e., sample root mass was multiplied by plot area and then divided by the sample area. Thus, root biomass represents an estimate of the total root mass per plot rather than a direct measurement of the entire topsoil root system. Because the same extrapolation constant was applied uniformly to all plots across generations, it does not affect comparisons among groups or generations.

Additionally, we included three registered cultivars per species as control groups to account for interannual environmental variation for the outdoor generations throughout the experiment. The purpose of including cultivar data in the analyzes was to assess to what extent changes in yield observed during the group selection experiment could be explained by variation in abiotic conditions across years (e.g., temperature and precipitation), rather than by the selection process itself by comparing the yield traits to that of stable and uniform cultivars. The cultivars were grown as part of variety testing field trials carried out annually by the Centre of Estonian Rural Research and Knowledge. The cultivars were Kalle, Kusta and Ivory for oat and Irina, Iron and Tuuli for barley. These cultivars are all commonly grown in Estonia. The cultivars were grown in close proximity to the group selection plots, on the same soil type, experiencing the same weather conditions and receiving the same amount of fertilizer. Only sowing density was different (500 germinating seeds per m^2^), as this is the recommended sowing density in Estonia. Data for grain mass (kg ha^−1^), seed humidity (%) and protein content (%) was collected from each cultivar.

Greenhouse generations were included in the experiment to speed up the selection process. However, since we were interested in yield gains under realistic field conditions, we focused data analysis and interpretation on the field generations.

### 2.2 Data Analysis

To assess variations in vegetative and generative traits across different generations of oat and barley, we constructed general linear models with various traits of interest (protein and grain yield, protein content, harvest index, plant height, elongation index, straw biomass, root mass, root length density, root:shoot ratio, root length ratio) included as dependent variables and generation included as a continuous independent variable. As many trait relationships were not linear, the quadratic form of the independent variable was included in models. Reflecting the different environmental conditions, analyzes were conducted separately for greenhouse and field generations. Because the summer generations were clearly affected by the amount of precipitation during the plant growth period, while greenhouse generations were affected by the amount of natural sunlight received in addition to the artificial illumination, these variables were included as covariates in the respective analyzes. Data on the amount of precipitation (mm) during the summer growth period and the total sunshine hours were obtained from the Estonian Weather Service.

As weather conditions and other factors, such as pathogen pressure, vary across years under field conditions, temporal yield trends in response to group selection can be masked by yield fluctuations due to environmental variation. To account for such interannual variation in growth conditions, we calculated a ln‐transformed ratio of yield (grain or protein) in each group selection plot to the average yield in the control groups (registered varieties of either oat or barley resulting from conventional breeding and grown under the same field conditions). The ratio is zero when there is no difference in yield between the group‐selected plants and the registered varieties, while more positive values indicate increased yield in group‐selected plots compared to control groups. We used general linear models to test trends in yield throughout the experiment relative to the registered cultivars. As with the analyzes of other trait variation described above, the models included both linear and quadratic terms for generation, while precipitation was included as a covariate to account for interannual environmental variation.

In addition, the average grain and protein yield of the first generation of the experiment grown in the target environment—in the field—was used as a benchmark to assess how yield changed throughout the selection process. For this analysis, the yield of each plot relative to the average yield of the first summer generation was calculated. General linear models were then carried out with yield ratio as the dependent variable, generation included as both linear and quadratic terms, and precipitation included as a covariate. However, it should be noted that the first summer generation did not represent the first generation of the experiment overall, as the experiment had initially begun with a greenhouse generation. Thus, the first summer generation represents the first generation grown in the target field environment and can be considered relatively unselected, although one cycle of selection had already occurred prior to it.

To test if group selection led to reduced trait variation between groups within each generation, Levene's tests were conducted for the field and greenhouse generations of each crop.

General linear models were fitted using the lm function from the R base package (version 3.6.1) (R Core Team 2019), and Levene's test was conducted using the leveneTest function from the car package in R (version 3.1–1) (Fox and Weisberg 2018).

## 3 Results

After eight generations, group‐selected oat and barley plots grown in the summer field generations produced significantly higher grain and protein yields relative to the first summer generation, which served as a minimally bred benchmark. This pattern was reflected by significant positive linear terms for both protein and grain yield ratios after accounting for precipitation (Table 1, Figure 2A,B). However, in both species and for both yield traits, the quadratic term was also significant and negative (Table 1, Figure 2A,B), indicating that although yield initially increased across generations, the rate of increase potentially has slowed over time and may have begun to plateau or decline in the final generations.

**TABLE 1:** Trends in yield traits expressed as yield ratios, calculated as plot yield divided by the average yield of the relevant control group.

	Oat			Barley		
	Estimated coefficient (SE)	*R* ^2^	*p*	Estimated coefficient (SE)	*R* ^2^	*p*
**(A) First group‐selected summer generation as control**						
*Protein yield (kg ha* ^*−1*^ *)*						
Linear term	2.35 × 10^−1^ (3.04 × 10^−2^)		**< 0.001**	2.36 × 10^−1^ (5.15 × 10^−2^)		**< 0.001**
Quadratic term	−5.16 × 10^−1^ (3.76 × 10^−2^)		**< 0.001**	−4.90 × 10^−1^ (6.24 × 10^−2^)		**< 0.001**
Precipitation (mm)	−3.01 × 10^−4^ (7.11 × 10^−5^)		**< 0.001**	−3.33 × 10^−4^ (1.21 × 10^−4^)		**0.009**
Whole‐model (df = 32)	0.91	**< 0.001**		0.74	**< 0.001**	
*Grain yield (kg ha* ^*−1*^ *)*						
Linear term	1.37 × 10^−1^ (3.47 × 10^−2^)		**< 0.001**	1.68 × 10^−1^ (4.72 × 10^−2^)		**0.001**
Quadratic term	−3.79 × 10^−1^ (4.28 × 10^−2^)		**< 0.001**	−2.28 × 10^−1^ (5.71 × 10^−2^)		**< 0.001**
Precipitation (mm)	−3.79 × 10^−1^ (8.10 × 10^−5^)		**0.012**	1.45 × 10^−5^ (1.11 × 10^−4^)		0.90
Whole‐model (df = 32)	0.92	**< 0.001**		0.64	**< 0.001**	
**(B) Registered cultivars as controls**						
*Protein yield (kg ha* ^*−1*^ *)*						
Linear term	5.88 × 10^−2^ (3.04 × 10^−2^)		0.06	1.29 × 10^−1^ (5.15 × 10^−2^)		**0.018**
Quadratic term	−4.76 × 10^−2^ (3.76 × 10^−2^)		0.21	−8.29 × 10^−2^ (6.24 × 10^−2^)		0.19
Precipitation (mm)	1.41 × 10^−4^ (7.11 × 10^−5^)		0.06	4.40 × 10^−5^ (1.21 × 10^−4^)		0.72
Whole‐model (df = 32)	0.05	0.20		0.33	**0.001**	
*Grain yield (kg ha* ^*−1*^ *)*						
Linear term	2.40 × 10^−2^ (3.47 × 10^−2^)		0.49	1.00 × 10^−1^ (4.72 × 10^−2^)		**0.042**
Quadratic term	5.82 × 10^−2^ (4.28 × 10^−2^)		0.18	1.20 × 10^−1^ (5.71 × 10^−2^)		**0.043**
Precipitation (mm)	−3.34 × 10^−5^ (8.10 × 10^−5^)		0.68	9.82 × 10^−5^ (1.11 × 10^−4^)		0.38
Whole‐model (df = 32)	0.13	0.06		0.31	**0.002**	

*Note:* In panel A, yields are compared with those of the first group‐selected generation grown in the target field environment, whereas in panel B, yields are compared with three concurrently grown registered cultivars. Due to significant non‐linearity for many traits, both linear and quadratic terms were included in the analysis. For plants growing outside, the main factor that varied from year to year was precipitation, and we included it as a covariate in the analysis. Estimated model coefficients and standard error values (in parentheses) are shown; significant *p*‐values are given in bold. In addition, the whole model goodness of the fit estimate (*R* ^2^) and its corresponding *p*‐value are also shown.

**FIGURE 2:**
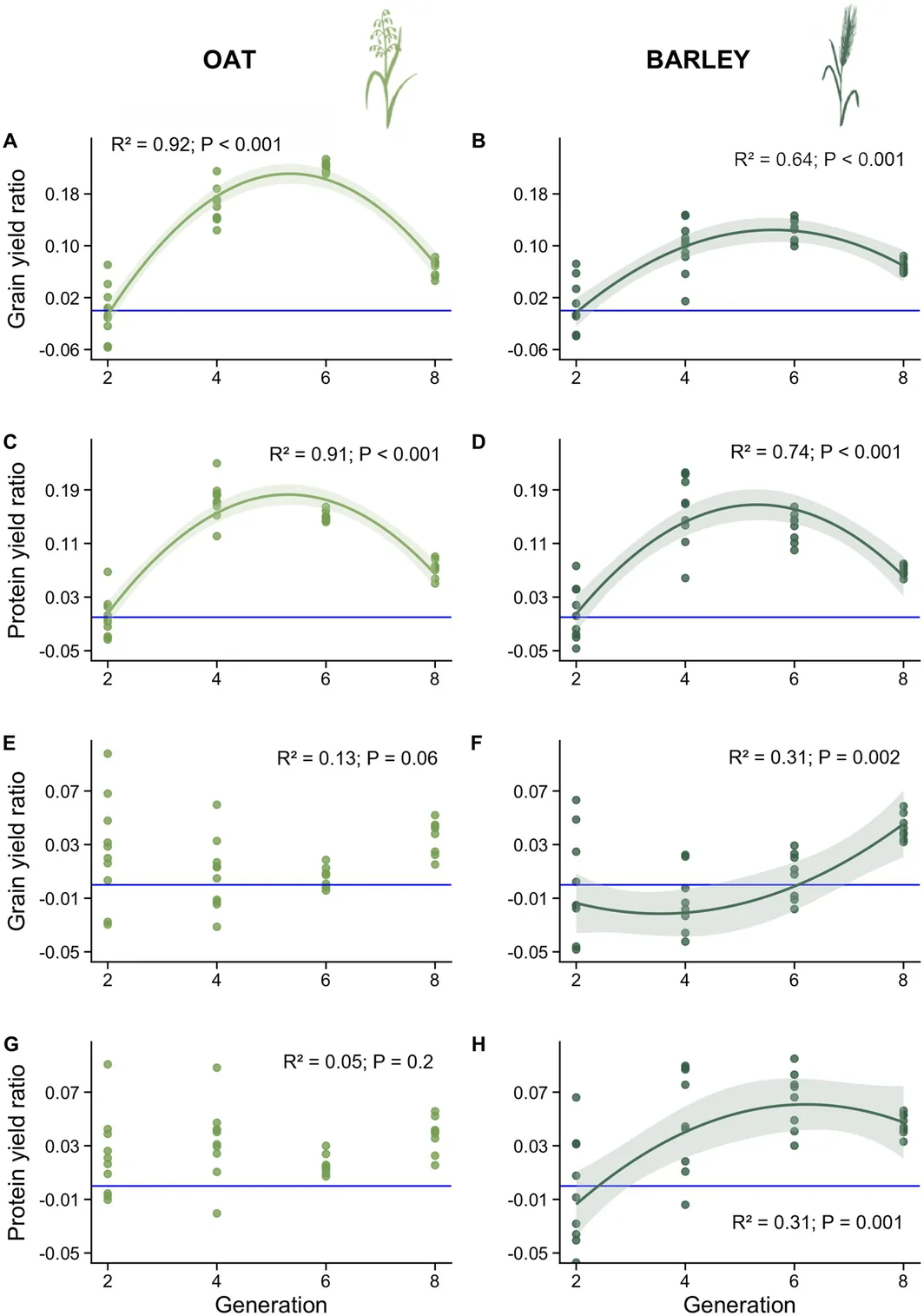
Grain and protein yield of oat and barley across eight generations of group selection, expressed relative to either the first summer generation of the group selection experiment (A–D) or concurrently grown registered cultivars (E–H). Values are shown as ln‐transformed yield ratios, calculated as the grain or protein yield of each group‐selected plot divided by the mean yield of the corresponding control group. The horizontal blue line at zero indicates no difference in yield between the group‐selected and control plants, whereas positive values indicate higher yield in the group‐selected plants. Solid lines represent significant fitted quadratic regression models and shaded areas denote their 95% confidence intervals. Whole‐model *R* ^2^ and *p*‐values are shown for each model. Estimated model coefficients, standard errors, and corresponding *p*‐values are presented in Table 1.

Registered cultivars were additionally included as stable and uniform control groups to account for environmental variation between generations. When yield ratios were calculated relative to these cultivars, no significant linear or quadratic terms were detected in oat. However, significant positive relationships for both grain and protein yield ratios were found in barley (Table 1, Figure 2C,D). This suggests that group selection may have increased yield performance in barley relative to registered cultivars grown concurrently under comparable environmental conditions. Sample sizes, mean yields, and confidence intervals of the mean for both the group‐selected plots and registered cultivars are presented in Table S1. It should be noted that yield in the intermediate winter generations grown in the greenhouse decreased in the course of the experiment (Table S3).

Alongside yield, the expression of several traits linked to investment into competitive organs gradually changed over the course of the experiment in summer generations (Figure 3, Tables S2, S5). Selection for maximum protein yield at the group level resulted in a significant decline in allocation to root growth. The reduction was detected both when root mass was analyzed as a standalone trait (Figure 3A,B; Table S2) and in comparison with allocation to straw biomass (Figure 3C,D).

**FIGURE 3:**
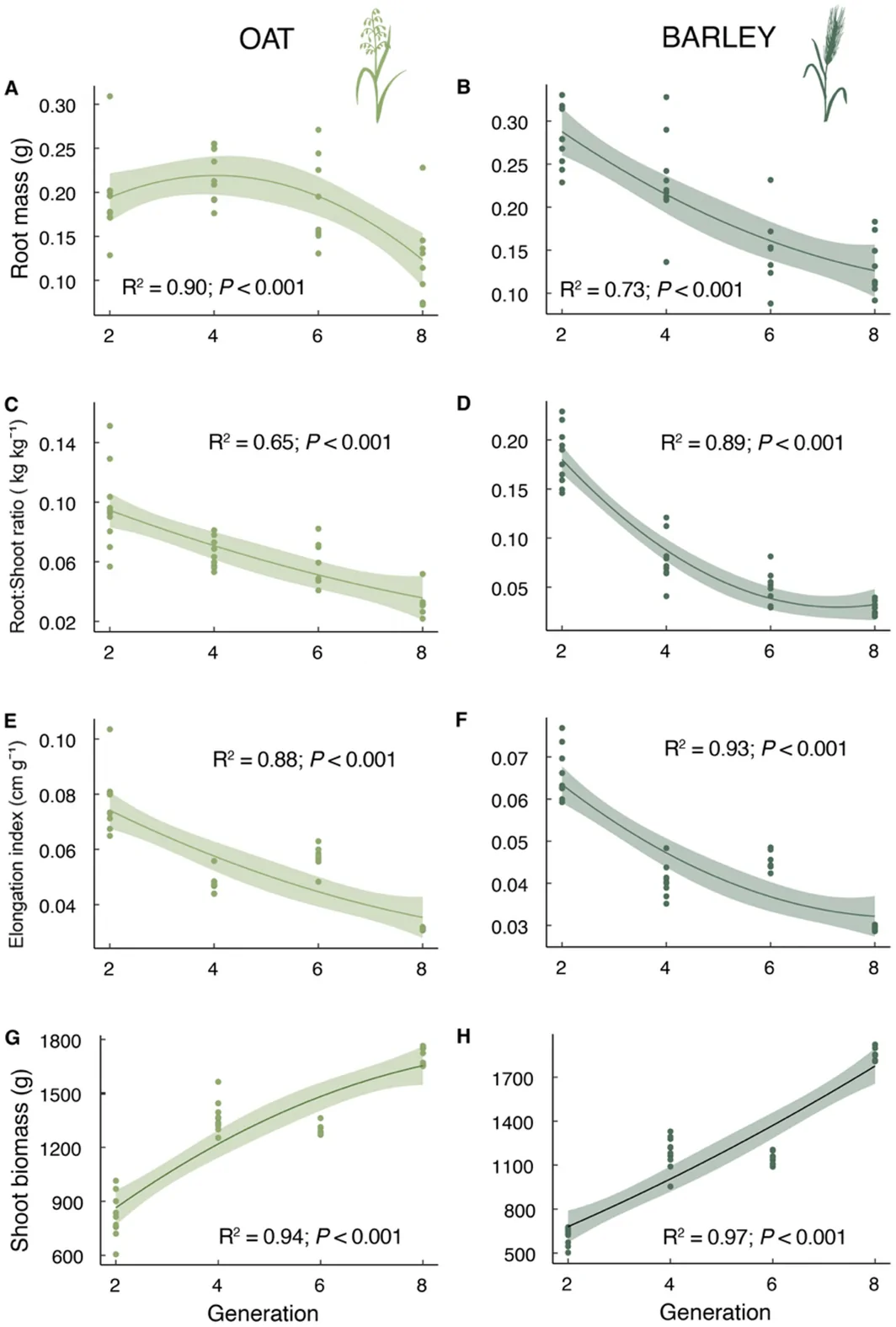
Changes in crop root growth and biomass allocation across eight generations of group selection. Variation across field‐grown generations of oat and barley is shown for the following traits: (A, B) Root mass; (C, D) Root dry mass to straw dry mass ratio; (E, F) Shoot elongation index (straw height to straw dry mass ratio); (G, H) Straw dry mass. Shaded areas denote 95% confidence intervals of the regression lines. Whole‐model *R* ^2^ and *p*‐values are shown.

Plant shoot elongation (plant height per unit of straw biomass) was another trait that gradually declined across summer generations in both oat and barley (Figure 3E,F; Table S2) while straw biomass and plant height both increased over the course of the experiment (Figure 3G,H, Tables S2, S5). Interestingly, trends in straw biomass and elongation index change across winter generations under greenhouse conditions were in the opposite direction to those detected under field conditions in summer generations (Table S4, Figure S2).

Throughout the experiment we detected considerable interannual variation in weather conditions; in particular, precipitation varied widely across years and significantly affected yield components as well as vegetative traits (Tables S2–S4; see climograph in Figure S1).

Group selection resulted in narrowing of yield and trait variation among groups with increasing numbers of generations under selection (Figures 2, 3, Figure S2; particularly in barley; Levene's test, Table S6), suggesting reduced genetic heterogeneity.

## 4 Discussion

### 4.1 Group Selection Was Associated With Increased Yield

By the end of the experiment, group‐selected oat and barley plants produced significantly higher grain and protein yields compared to the beginning of the experiment, although a significant negative quadratic term may suggest the yield gain is decelerating or turning. In barley, a similar positive yield gain trend was also detected when yield was compared with that of conventional registered cultivars grown concurrently under comparable environmental conditions, suggesting that the observed response was not solely driven by environmental conditions that naturally vary year‐by‐year, although environmental effects cannot be entirely ruled out either. In oat, however, the corresponding trend relative to conventional cultivars was not significant, which may indicate a stronger influence of abiotic environmental conditions on oat yield responses. Overall, this demonstrates that the group selection process can be applied to crosses of current cereal varieties and lead to improved yield. This also supports the predictions of previous studies that yield can be further improved by targeting traits that improve group‐level rather than individual performance due to the existence of a genetically based trade‐off between high yield in a genetically homogeneous group (corresponding to high group‐level yield) and individual performance when competing with genetically diverse neighbors (Acquaah 2012; Goodnight et al. 1992). However, it must be noted that with the current experimental design we cannot conclusively determine whether the observed effects were specifically attributable to group selection or other processes such as population segregation, heterosis or a combination of these factors. Furthermore, the intermediate winter generations grown in the greenhouse showed yield as well as some vegetative trends opposite to those observed in the summer generations. Winter generations were included in the experimental design primarily to speed up the selection process. The absence of similar trends under greenhouse conditions suggests that phenotypic plasticity and genotype by environment interactions contributed to the observed responses. However, it should also be noted that the greenhouse environment differed significantly from the field environment: plants were grown in boxes containing a different peat‐based substrate and received additional artificial lighting, as during winters there is low availability of natural light in the region. Selection plausibly changed the genetic architecture, but trait expression may still be environment‐dependent, potentially suggesting gene–environment interaction.

### 4.2 Selection for Group‐Level Yield Reduced Investment Into Competitive Traits

In our experiment, no traits besides protein yield at the group level were used to inform breeding, yet by the end of the experiment, there were clear changes in plant architecture and canopy structure. Group‐level straw biomass increased, root biomass allocation decreased, and thus also the root:shoot ratio decreased over the course of the experiment, indicating that the group selection method led to more efficient biomass allocation to resource‐acquiring organs and conversion of acquired resources to grain production. Roots have never received specific attention in the breeding of cereal crops, and changes in root architecture during the course of conventional plant breeding history have primarily occurred as an incidental effect of selection for higher crop yield (Anten and Vermeulen 2016). It has been proposed that breeding crops with smaller root systems, which decreases excessive root production in response to competitors, could result in higher yield (Zhu et al. 2018; Gersani et al. 2001; Zhu and Zhang 2013), particularly given that root production is about twice as costly per biomass unit as the production of shoots (Passioura 1983). Furthermore, most crop breeding is conducted under relatively fertile soil conditions, and the majority of crops are grown in agricultural systems where nutrients are supplemented through fertilization (Acquaah 2012; FAO 2024; Atlin and Frey 1989). This further reduces the need for plants to invest in extensive root systems for nutrient acquisition. Indeed, recent research has uncovered a trade‐off between root production and yield across existing cereal cultivars—greater investment into root systems is associated with lower grain yield (Vain et al. 2023; Feng et al. 2023). Our study provides direct evidence for persisting excessive root growth in modern crops, especially in the topsoil, which we were able to reduce by targeting group‐level grain protein yield. However, reduced allocation to root biomass may not be universally advantageous under all environmental conditions. For example, it is well established that greater investment in root mass and the development of longer roots can improve yield under drought conditions (Siddiqui et al. 2021). In our experiment, the average rainfall during the growing season was below the long‐term regional average in all years except 2020 (corresponding to generation 6). While reduced rainfall does not necessarily constitute drought, these conditions still suggest that reduced root biomass in group‐selected plants did not result in significant yield penalties, even under suboptimal water availability.

It is also commonly argued that under drought, the production of deeper roots is particularly beneficial (Siddiqui et al. 2021). However, since our root sampling was limited to the topsoil, we do not have data on rooting depth and therefore cannot draw conclusions on whether group selection influenced this trait.

Plant shoot elongation also gradually declined despite not being a direct target of selection in our experiment. In general, plant elongation in dense stands is key to success in competition for light, as taller plants are better able to shade and thus suppress their neighbors. However, while elongation benefits individual fitness, the trade‐off between plant height and crop yield is well known (Law et al. 1978), and ever since the Green Revolution in the 1960s, plant height has declined in breeding programs (Würschum et al. 2017). The main motivation for this has been to improve lodging resistance and increase harvest index (yield to aboveground biomass ratio), but this may have resulted in suboptimal heights for achieving greatest yield benefits via efficient utilization of light resources within plant canopies (Slattery and Ort 2021). Interestingly, shoot elongation decreased, but overall straw mass as well as plant height increased. Hence, group selection enabled further improvements in crop architecture—plants produced denser and sturdier canopies above ground. Such a shift in allocation should help plants support heavier spikes and better resist lodging, as sturdier plants are generally more resistant to lodging (Dockter and Hansson 2015).

### 4.3 The Challenge of Selection Under Fluctuating Environmental Conditions

This experiment involved selection during several years, with fluctuating weather conditions, both in the field and in the greenhouse during the winter generations. In addition, contrasting environmental conditions in the field versus greenhouse may have created opposing selective pressures on plants in alternating generations. Temperature and humidity were higher in the greenhouse, soil conditions differed, and while plants received additional artificial illumination, the light levels were lower than those in the field during summer. Indeed, different trends in yield and vegetative traits were detected under greenhouse conditions, highlighting that selection outcomes and plant–plant interactions, competitive or cooperative, are context‐dependent (Zhang and Tielbörger 2020) and cannot be easily translated from greenhouse studies to realistic field conditions. We therefore focused primarily on the outcome of selection under typical agronomic practices in the field. In addition, observed gradual changes in traits related to crop competitive ability across generations suggest the group selection methodology can produce consistent outcomes even in the presence of environmental fluctuations.

### 4.4 Recommendations for Future Group Selection Studies

There are some limitations in the study design that prevent us from drawing strong conclusions whether the effects seen can be attributed to group selection or whether there is some other genetic effect, e.g., heterosis, in play. In particular, we used conventionally bred varieties grown in proximity of experimental plots as a control for potential interannual variation in crop yields that is independent of our selection treatment, as well as the first field generation of plants as a control to test changes in yield traits of group‐selected plants throughout the experiment. A better way to test if our results are indeed driven by group selection would be to construct control groups from the same starting genetic material that would be propagated either in the absence of group selection (a random group going forward to the next generation), in the presence of individual selection or selection for low protein yield at various sowing densities. This could enable us to test whether changes in yield can be ascribed to segregating populations (group selected plants), interplant interactions or to some environmental conditions other than the amount of precipitation which was used as a covariate in the analyzes. Furthermore, increasing the number of generations and groups in future studies could help ensure that the observed effects result from directed evolution rather than random, unpredictable variation. As genetic diversity within groups naturally decreases over successive generations in segregating populations, extending the experiment would also help to confirm the hypothesis that the observed effects are indeed due to the selection of cooperative genotypes.

In this experiment, original crosses were used as the starting material, which inherently are genetically diverse. This genetic and associated phenotypic diversity benefits a selection experiment by increasing the chances of cooperative genotypes being present in the population, which can then be identified and sifted out through group selection. However, this creates a situation where the genetic diversity within groups is comparable to diversity between groups, while effective group selection requires low genetic diversity within groups. It has been shown in modeling simulations that group selection is most effective when groups are composed of closely related and hence phenotypically similar individuals (Montazeaud et al. 2020), Therefore, in future studies, we recommend applying group selection to a wide array of crosses to increase the likelihood of detecting the genotypes with the greatest potential for high group yield, but to assemble initial groups from closely related rather than randomly selected individuals (e.g., siblings of a single mother plant).

Although we started with high genetic diversity within groups, selection resulted in narrowing of yield and trait variation across generations suggesting reduced genetic heterogeneity. Reduced trait variation in the later generations could have contributed to more effective selection for group‐level yield compared with earlier generations. In the early stages of group selection, competitive genotypes may have gained dominance within genetically heterogeneous groups, reducing the potential of less competitive genotypes to enhance group‐level yield. This may have contributed to the observed transient declines in yield during intermediate generations (Figure S2). While group selection promotes the evolution of cooperative traits, it does not necessarily eliminate within‐group competition; rather, the balance may shift toward cooperation becoming more predominant. Thus, some level of competition likely persists even in groups selected for cooperative performance.

### 4.5 Group Selection Targets Beyond Reducing Competitive Behavior

Despite the limitations of the experimental design and the need for additional control groups, our study provides the first empirical evidence for the potential of group‐level selection to identify genotypes with traits that enhance crop yield at the group rather than individual level. This approach links selection outcomes with typical agronomic objective, which is to maximize field‐level yield. We found that selection for higher group‐level yield resulted in gradual shifts towards reduced root system size and sturdier, less elongated plants above ground, highlighting the traits that could be the target of selection for enhanced group‐level yields. These are complex traits that can reflect multiple changes in crop morphology and physiology, as well as changes in plant‐microbial interactions. For example, group‐level yield can be enhanced by not only reducing traits that regulate competitive interference (shading and nutrient contest) but also by enhancing traits that enable plants to maintain high levels of grain production despite resource shortage in densely sown crop stands (i.e., traits underlying competitive tolerance, such as shade tolerance). Below ground such traits may include efficient spatial positioning of roots in soil (Cabal et al. 2020; Semchenko 2020), reduced allelopathic responses (Kong et al. 2018) and enhanced associations with arbuscular mycorrhizal fungi, which colonize plant roots and can supply nutrients at a lower cost to the plant than producing an extensive root system (Bergmann et al. 2020). Group selection may therefore indirectly select for traits that enable nutrient uptake with smaller root systems, such as via mycorrhizal associations. Microbial symbionts have become redundant under modern agricultural practices with high fertilizer and pesticide inputs (Porter and Sachs 2020) but are becoming a focus of breeding with the aim of alleviating the environmental impacts of agriculture (Harman et al. 2021). We propose that group selection may also be a promising tool for maximizing the benefit of such microbe‐centered breeding approaches.

## Funding

This work was supported by Estonian Research Council grant (PRG 1223, PRG 1065), Estonian Ministry of Education and Research (Center of Excellence AgroCropFuture “Agroecology and new crops in future climates,” TK200).

## Conflicts of Interest

The authors declare no conflicts of interest.

## Supporting information


**Table S1:** Sample sizes, mean grain and protein yield values, and lower and upper 95% confidence interval limits for oat and barley summer generations. Values are shown for both the group‐selected plots and the three registered cultivars used as controls.
**Table S2:** Changes in plant traits during the course of eight generations in a group selection experiment. Models include data from summer generations (2, 4, 6, 8) grown under field conditions. Due to significant non‐linearity in many traits, generation was included in the analyzes as linear and quadratic terms. For plants growing outside the main factor that varied from year to year was precipitation, and we included it as a covariate in analyzes. Estimated model coefficients and their standard errors (in parentheses) are shown, significant *p*‐values are given in bold (*p* < 0.05). In addition, the whole model goodness of the fit estimate (*R* ^2^) and its corresponding *p*‐value are also shown.
**Table S3:** Changes in generative plant traits during the course of eight generations in a group selection experiment. Models include data from winter generations (1, 3, 5, 7) grown in the greenhouse. Due to significant non‐linearity in many traits, generation was included in the analyzes as linear and quadratic terms. For plants growing indoors in winter, the main factor that varied from year to year was the amount of sunlight (in addition to artificial illumination), and we included it as a covariate in analyzes. Estimated model coefficients and standard error values (in parentheses) are shown, significant *p*‐values are given in bold. In addition, the whole model goodness of the fit estimate (*R* ^2^) and its corresponding *p*‐value are also shown.
**Table S4:** Changes in vegetative plant traits during the course of eight generations in a group selection experiment. Models include data from winter generations (1, 3, 5, 7) grown in the greenhouse. Due to significant non‐linearity in many traits, generation was included in the analyzes as linear and quadratic terms. For plants growing indoors in winter, the main factor that varied from year to year was the amount of sunlight (in addition to artificial illumination), and we included it as a covariate in analyzes. Estimated model coefficients and standard error values (in parentheses) are shown, significant *p*‐values are given in bold. In addition, the whole model goodness of the fit estimate (*R* ^2^) and its corresponding *p*‐value are also shown.
**Table S5:** Changes in various traits in the course of group selection summer generations in oat and barley. Due to significant non‐linearity for many traits, both linear and quadratic terms were included in the analysis. For plants growing outside, the main factor that varied from year to year was precipitation, and we included it as a covariate in the analysis. Estimated model coefficients and standard error values (in parentheses) are shown, significant *p*‐values are given in bold. In addition, the whole model goodness of the fit estimate (*R* ^2^) and its corresponding *p*‐value are also shown.
**Table S6:** Levene's test results for various traits in oat and barley over the summer generations. Statistical significance indicates that there exists a significant difference in within‐trait variation between the groups compared.
**Figure S1:** A climograph showing the average temperature (line) and total precipitation (bars) during the period form sowing until maturity in each field season of the group selection experiment (from 2018 to 2021).
**Figure S2:** Changes in crop root growth and biomass allocation for winter generations across eight generations of group selection. Variation across group selected oat and barley are shown for the following traits: (A, B) Root mass; (C, D) Root dry mass to shoot dry mass ratio; (E, F) Shoot elongation index (straw height to shoot dry mass ratio); (G, H) Shoot dry mass. Shaded areas denote confidence 95% confidence intervals of the regression lines. Whole‐model *R* ^2^ and *p*‐values are shown. For significance estimates for quadratic and linear terms, see Table S2. Note that the experiment comprised winter (1, 2, 5, 7) and summer (2, 4, 6, 8) generations; only the winter generations, which experienced greenhouse conditions are shown.

## Data Availability

Data underlying this experiment is available on Figshare (https://doi.org/10.6084/m9.figshare.25433953).
