# Supplementary material for: Group Selection has the Potential to Increase Yield and Resource Use Efficiency in Cereal Crops
Source: Evol Appl. 2026 Nov 11;19(11):e70347. doi: 10.1111/eva.70347 (PMC13755528; doi:10.1111/eva.70347)
Supplement: Supplementary file 1 [file EVA-19-e70347-s001.docx]

**Table S1.** Sample sizes, mean grain and protein yield values, and lower and upper 95% confidence interval limits for oat and barley summer generations. Values are shown for both the group-selected plots and the three registered cultivars used as controls.

|  | | **Oat** | | | | **Barley** | | | |
| --- | --- | --- | --- | --- | --- | --- | --- | --- | --- |
|  |  | **Grain yield (kg ha^-1^)** | | **Protein yield (kg ha^-1^)** | | **Grain yield (kg ha^-1^)** | | **Protein yield (kg ha^-1^)** | |
|  |  | Group selection | Cultivar controls | Group selection | Cultivar controls | Group selection | Cultivar controls | Group selection | Cultivar controls |
| **Generation 2** | n | 10 | 3 | 10 | 3 | 10 | 3 | 10 | 3 |
|  | mean | 3624.5 | 3404.2 | 441.6 | 418.7 | 3999.9 | 4082.1 | 447.5 | 458.3 |
|  | CI (lower, upper) | 3385.8, 3863.2 | 3012.8, 3795.5 | 418.7, 464.5 | 360.9, 476.5 | 3670.8, 4328.9 | 3667.7, 4496.5 | 412.3, 482.8 | 389.7, 526.9 |
| **Generation 4** | n | 10 | 3 | 10 | 3 | 10 | 3 | 10 | 3 |
|  | mean | 5276.8 | 5182.1 | 661.7 | 611.4 | 5032.5 | 5336.9 | 656.5 | 598.6 |
|  | CI (lower, upper) | 5043.7, 5509.9 | 4818.1, 5546.0 | 631.4, 691.9 | 468.1, 754.8 | 4708.0, 5357.0 | 5094.8, 5578.9 | 602.9, 710.2 | 527.0, 670.2 |
| **Generation 6** | n | 8 | 3 | 8 | 3 | 8 | 3 | 8 | 3 |
|  | mean | 6015.4 | 5945.5 | 624.8 | 602.3 | 5327.3 | 5240.8 | 610.0 | 525.6 |
|  | CI (lower, upper) | 5922.1, 6108.7 | 5733.0, 6157.9 | 615.7, 634.0 | 538.9, 665.8 | 5148.6, 5505.9 | 4567.4, 5914.3 | 584.0, 636.0 | 469.3, 581.9 |
| **Generation 8** | n | 8 | 3 | 8 | 3 | 8 | 3 | 8 | 3 |
|  | mean | 4223.5 | 3891.2 | 522.1 | 478.3 | 4684.8 | 4243.8 | 525.8 | 472.6 |
|  | CI (lower, upper) | 4118.4, 4328.7 | 3309.8, 4472.6 | 508.6, 535.7 | 422.6, 534.0 | 4599.0, 4770.5 | 3371.5, 5116.1 | 517.8, 533.7 | 370.2, 574.9 |

**Table S2.** Changes in plant traits during the course of eight generations in a group selection experiment. Models include data from summer generations (2, 4, 6, 8) grown under field conditions. Due to significant non-linearity in many traits, generation was included in the analyses as linear and quadratic terms. For plants growing outside the main factor that varied from year to year was precipitation, and we included it as a covariate in analyses. Estimated model coefficients and their standard errors (in parentheses) are shown, significant *P*-values are given in bold (*P* < 0.05). In addition, the whole model goodness of the fit estimate (R^2^) and its corresponding *P*-value are also shown.

|  | Oat | | | Barley | | |
| --- | --- | --- | --- | --- | --- | --- |
|  | Estimated coefficient (SE) | R^2^ | *P* | Estimated coefficient (SE) | R^2^ | *P* |
| **Protein yield (kg ha^-1^**) | | | | | | |
| Linear term | 2.71*10^2^ (3.92*10^1^) | | **<0.001** | 2.74*10^2^ (66.1) | | **<0.001** |
| Quadratic term | -6.60*10^2^ (4.83*10^1^) | | **<0.001** | -6.35*10^2^ (80.0) | | **<0.001** |
| Precipitation (mm) | 3.94*10^-1^ (9.15*10^-2^) | | **<0.001** | 4.49*10^-1^ (1.55*10^-1^) | | **0.007** |
| Whole-model (df = 32) | | 0.90 | **<0.001** |  | 0.73 | **<0.001** |
| **Root mass (g)** | | | | | | |
| Linear term | -1.36*10^-1^ (5.87*10^-2^) | | **0.027** | -2.99*10^-1^ (5.62*10^-2^) | | **<0.001** |
| Quadratic term | -1.68*10^-1^ (7.24*10^-2^) | | **0.027** | -3.40*10^-2^ (6.80*10^-2^) | | 0.62 |
| Precipitation (mm) | -5.31*10^-5^ (1.37*10^-4^) | | 0.70 | 2.26*10^-4^ (1.32*10^-4^) | | 0.10 |
| Whole-model (df = 32) | | 0.36 | **<0.001** |  | 0.68 | **<0.001** |
| **Root:Shoot ratio (kg kg^-1^)** | | | | | | |
| Linear term | -4.22*10^-4^ (5.85*10^-5^) | | **<0.001** | -9.42*10^-4^ (7.16*10^-5^) | | **<0.001** |
| Quadratic term | 1.91*10^-4^ (7.22*10^-5^) | | 0.11 | 4.68*10^-4^ (8.66*10^-5^) | | **<0.001** |
| Precipitation (mm) | 2.49*10^-7^ (1.37*10^-7^) | | 0.07 | 3.57*10^-7^ (1.68*10^-7^) | | **0.041** |
| Whole-model (df = 32) | | 0.65 | **<0.001** |  | 0.89 | **<0.001** |
| **Shoot biomass (g m^-2^)** | | | | | | |
| Linear term | 2.41*10^3^ (1.10*10^2^) | | **<0.001** | 3.22*10^3^ (9.52*10^1^) | | **<0.001** |
| Quadratic term | -1.23*10^3^ (1.36*10^2^) | | **<0.001** | -1.01*10^3^ (1.15*10^2^) | | **<0.001** |
| Precipitation (mm) | -2.29 (2.58*10^-1^) | | **<0.001** | -2.81 (2.23*10^-1^) | | **<0.001** |
| Whole-model (df = 32) | | 0.94 | **<0.001** |  | 0.97 | **<0.001** |
| **Elongation index (cm/g)** | | | | | | |
| Linear term | -1.30*10^-1^ (8.34*10^-3^) | | **<0.001** | -9.93*10^-2^ (5.04*10^-3^)  5.89*10^-2^ (6.10*10^-3^)  1.03*10^-4^ (1.18*10^-5^) | | **<0.001** |
| Quadratic term | 7.48*10^-2^ (1.03*10^-2^) | | **<0.001** |  |  | **<0.001** |
| Precipitation (mm) | 1.53*10^-4^ (1.95*10^-5^) | | **<0.001** |  |  | **<0.001** |
| Whole-model (df = 32) | | 0.88 | **<0.001** |  | 0.93 | **<0.001** |

**Table S3.** Changes in generative plant traits during the course of eight generations in a group selection experiment. Models include data from winter generations (1, 3, 5, 7) grown in the greenhouse. Due to significant non-linearity in many traits, generation was included in the analyses as linear and quadratic terms. For plants growing indoors in winter, the main factor that varied from year to year was the amount of sunlight (in addition to artificial illumination), and we included it as a covariate in analyses. Estimated model coefficients and standard error values (in parentheses) are shown, significant P-values are given in bold. In addition, the whole model goodness of the fit estimate (R^2^) and its corresponding P-value are also shown.

|  | Oat | | | Barley | | |
| --- | --- | --- | --- | --- | --- | --- |
|  | Estimated  coefficient (SE) | R^2^ | P-value | Estimated  coefficient (SE) | R^2^ | p |
| **Protein yield (kg ha^-1^**) | |  |  |  |  |  |
| Linear term | -2.99*10^2^ (5.20*10^1^) |  | **<0.001** | -9.45*10^-2^ (5.21*10^1^) |  | **<0.001** |
| Quadratic term | -1.62*10^2^ (2.96*10^1^) |  | **<0.001** | 2.60*10^2^ (2.67*10^1^) |  | **<0.001** |
| Sunlight hours (h) | -1.43*10^-1^ (6.96*10^-2^) |  | **0.048** | -8.56*10^-1^ (7.00*10^-2^) |  | **<0.001** |
| Whole-model (df = 32) | | 0.73 | **<0.001** |  | 0.95 | **<0.001** |
| **Grain yield (kg ha^-1^)** | |  |  |  |  |  |
| Linear term | -2.56*10^3^ (4.15*10^2^) |  | **<0.001** | -5.35*10^3^ (4.49*10^2^) |  | **<0.001** |
| Quadratic term | -1.38*10^3^ (2.36*10^2^) |  | **<0.001** | 8.11*10^2^ (2.31*10^2^) |  | **0.001** |
| Sunlight hours (h) | -2.16 (5.55*10^-1^) |  | **<0.001** | -6.09 (6.04*10^-1^) |  | **<0.001** |
| Whole-model (df = 32) | | 0.65 | **<0.001** |  | 0.85 | **<0.001** |
| **Harvest Index (kg/kg)** | |  |  |  |  |  |
| Linear term | -3.84*10^-2^ (2.20*10^-2^) |  | 0.09 | -3.55*10^-2^ (2.52*10^-2^) |  | 0.17 |
| Quadratic term | -1.32*10^-1^ (1.25*10^-2^) |  | **<0.001** | 2.26*10^-2^ (1.29*10^-2^) |  | 0.09 |
| Sunlight hours (h) | -5.89*10^-5^ (2.95*10^-5^) |  | 0.05 | -9.18*10^-5^ (3.39*10^-5^) |  | **0.011** |
| Whole-model (df = 32) | | 0.78 | **<0.001** |  | 0.32 | **0.001** |
| **Protein content (%)** | |  |  |  |  |  |
| Linear term | -1.88*10^-1^ (7.17*10^-1^) |  | 0.79 | -7.66 (6.69*10^-1^) |  | **<0.001** |
| Quadratic term | -1.36*10^-1^ (4.07*10^-1^) |  | 0.74 | 3.55 (3.44*10^-1^) |  | **<0.001** |
| Sunlight hours (h) | 2.34*10^-3^ (9.59*10^-4^) |  | **0.021** | -4.44*10^-3^ (9.00*10^-4^) |  | **<0.001** |
| Whole-model (df = 32) | | 0.46 | **<0.001** |  | 0.92 | **<0.001** |

**Table S4.** Changes in vegetative plant traits during the course of eight generations in a group selection experiment. Models include data from winter generations (1, 3, 5, 7) grown in the greenhouse. Due to significant non-linearity in many traits, generation was included in the analyses as linear and quadratic terms. For plants growing indoors in winter, the main factor that varied from year to year was the amount of sunlight (in addition to artificial illumination), and we included it as a covariate in analyses. Estimated model coefficients and standard error values (in parentheses) are shown, significant P-values are given in bold. In addition, the whole model goodness of the fit estimate (R^2^) and its corresponding P-value are also shown.

|  | Oat | | | Barley | | |
| --- | --- | --- | --- | --- | --- | --- |
|  | Estimated  coefficient (SE) | R^2^ | P-value | Estimated  coefficient (SE) | R^2^ | p |
| **Root mass (g)** | |  |  |  |  |  |
| Linear term | -7.88*10^-2^ (5.35*10^-2^) |  | 0.15 | -7.02*10^-2^ (5.22*10^-2^) |  | 0.19 |
| Quadratic term | -1.91*10^-3^ (3.04*10^-2^) |  | 0.95 | 7.54*10^-2^ (2.67*10^-2^) |  | **0.008** |
| Sunlight hours (h) | -6.06*10^-5^ (7.15*10^-5^) |  | 0.40 | -3.50*10^-5^ (7.02*10^-5^) |  | 0.62 |
| Whole-model (df = 32) | | 0.003 | 0.39 |  | 0.24 | **0.008** |
| **Root length density (cm cm^-3^)** | |  |  |  |  |  |
| Linear term | -1.41 (5.97*10^-1^) |  | **0.024** | -1.81 (7.82*10^-1^) |  | **0.027** |
| Quadratic term | -4.93*10^-3^ (3.39*10^-1^) |  | 0.99 | 8.18*10^-1^ (4.01*10^-1^) |  | 0.05 |
| Sunlight hours (h) | -1.25*10^-3^ (7.99*10^-4^) |  | 0.13 | -8.63*10^-4^ (1.05*10^-3^) |  | 0.42 |
| Whole-model (df = 32) | | 0.11 | 0.08 |  | 0.30 | **0.002** |
| **Root:Shoot ratio (kg kg^-1^)** | |  |  |  |  |  |
| Linear term | -3.74*10^-5^ (3.55*10^-5^) |  | 0.29 | -3.26*10^-6^ (3.74*10^-5^) |  | 0.93 |
| Quadratic term | -2.19*10^-5^ (2.02*10^-5^) |  | 0.30 | 5.24*10^-5^ (1.92*10^-5^) |  | **0.010** |
| Sunlight hours (h) | -3.58*10^-8^ (4.74*10^-8^) |  | 0.46 | 1.23*10^-8^ (5.03*10^-8^) |  | 0.81 |
| Whole-model (df = 32) | | 0.03 | 0.60 |  | 0.13 | 0.06 |
| **Root length ratio (m kg^-1^)** | |  |  |  |  |  |
| Linear term | -1.05 (5.50*10^-1^) |  | 0.06 | -1.44 (6.3*10^-1^) |  | **0.031** |
| Quadratic term | -3.22*10^-1^ (3.13*10^-1^) |  | 0.31 | 8.10*10^-1^ (3.25*10^-1^) |  | **0.018** |
| Sunlight hours (h) | 1.09*10^-3^ (7.36*10^-4^) |  | 0.15 | 5.08*10^-4^ (8.46*10^-4^) |  | 0.55 |
| Whole-model (df=32) | | 0.03 | 0.29 |  | 0.34 | **0.001** |
| **Shoot biomass (g m^-2^)** | |  |  |  |  |  |
| Linear term | -6.34*10^-2^ (1.57*10^2^) |  | **<0.001** | -1.33*10^3^ (1.22*10^2^) |  | **<0.001** |
| Quadratic term | 6.64*10^2^ (8.92*10^1^) |  | **<0.001** | 1.77*10^2^ (6.24*10^1^) |  | **0.008** |
| Sunlight hours (h) | -3.14*10^-1^ (2.10*10^-1^) |  | 0.14 | -1.05 (1.64*10^-1^) |  | **<0.001** |
| Whole-model (df = 32) | | 0.77 | **<0.001** |  | 0.84 | **<0.001** |
| **Plant height (cm**) | |  |  |  |  |  |
| Linear term | 5.79 (1.22*10^1^) |  | 0.64 | 2.38*10^1^ (7.57) |  | **0.004** |
| Quadratic term | -2.49*10^1^ (6.93) |  | **0.001** | 3.86*10^1^ (3.88) |  | **<0.001** |
| Sunlight hours (h) | -5.31*10^-2^ (1.63*10^-2^) |  | **0.003** | 9.98*10^-3^ (1.02*10^-2^) |  | 0.33 |
| Whole-model (df = 32) | | 0.62 | **<0.001** |  | 0.78 | **<0.001** |
| **Elongation index (cm/g)** | |  |  |  |  |  |
| Linear term | 3.41*10^-2^ (1.24*10^-2^) |  | **0.010** | 9.43*10^-2^ (1.09*10^-2^) |  | **<0.001** |
| Quadratic term | -6.51*10^.2^ (7.07*10^-3^) |  | **<0.001** | 1.46*10^-2^ (5.62*10^-3^) |  | **0.014** |
| Sunlight hours (h) | -3.00*10^-5^ (1.66*10^-5^) |  | 0.08 | 7.67*10^-5^ (1.47*10^-5^) |  | **<0.001** |
| Whole-model (df = 32) | | 0.83 | **<0.001** |  | 0.73 | **<0.001** |

**Table S5.** Changes in various traits in the course of group selection summer generations in oat and barley. Due to significant non-linearity for many traits, both linear and quadratic terms were included in the analysis. For plants growing outside, the main factor that varied from year to year was precipitation, and we included it as a covariate in the analysis. Estimated model coefficients and standard error values (in parentheses) are shown, significant P-values are given in bold. In addition, the whole model goodness of the fit estimate (R^2^) and its corresponding P-value are also shown.

|  | Oat | | | | Barley | | |
| --- | --- | --- | --- | --- | --- | --- | --- |
|  | Estimated coefficient (SE) | | R^2^ | P-value | Estimated coefficient (SE) | R^2^ | p |
| **Grain yield (kg ha^-1^)** | | |  |  |  |  |  |
| Linear term | | 1.01*10^3^ (3.43*10^2^) |  | **0.006** | 1.58*10^3^ (4.76*10^2^) |  | **0.002** |
| Quadratic term | | -3.66*10^3^ (4.24*10^2^) |  | **<0.001** | -2.33*10^3^ (5.76*10^2^) |  | **<0.001** |
| Precipitation (mm) | | 3.33 (8.03*10^-1^) |  | **<0.001** | 4.12*10^-1^ (1.12) |  | 0.71 |
| Whole-model (df = 32) | | | 0.93 | **<0.001** |  | 0.66 | **<0.001** |
| **Plant height (cm**) | | |  |  |  |  |  |
| Linear term | 2.26*10^1^ (4.56) | |  | **<0.001** | 2.14*10^1^ (2.42) |  | **<0.001** |
| Quadratic term | -4.68*10^1^ (5.62) | |  | **<0.001** | -2.11 (2.93) |  | 0.48 |
| Precipitation (mm) | 3.92*10^-2^ (1.07*10^-2^) | |  | **<0.001** | -1.69*10^-3^ (5.68*10^-3^) |  | 0.77 |
| Whole-model (df = 32) | | | 0.93 | **<0.001** |  | 0.78 | **<0.001** |
| **Root length ratio (m kg^-1^)** | | |  |  |  |  |  |
| Linear term | -4.20 (0.58) | |  | **<0.001** | -6.97 (8.46*10^-1^) |  | **<0.001** |
| Quadratic term | -1.02 (0.71) | |  | 0.16 | -2.97*10^-1^ (1.02) |  | 0.77 |
| Precipitation (mm) | -6.71*10^-4^ (1.33*10^-3^) | |  | 0.62 | 2.98*10^-3^ (1.98*10^-3^) |  | 0.14 |
| Whole-model (df=32) | | | 0.75 | **<0.001** |  | 0.81 | **<0.001** |
| **Root length density (cm cm^-3^)** | | |  |  |  |  |  |
| Linear term | -4.16*10^-1^ (5.34*10^-1^) | |  | 0.44 | -1.12 (5.16*10^-1^) |  | **0.038** |
| Quadratic term | -4.21 (6.59*10^-1^) | |  | **<0.001** | -4.03 (6.24*10^-1^) |  | **<0.001** |
| Precipitation (mm) | -4.19*10^-3^ (1.25*10^-3^) | |  | **0.002** | -6.75*10^-3^ (1.21*10^-3^) |  | **<0.001** |
| Whole-model (df = 32) | | | 0.64 | **<0.001** |  | 0.74 | **<0.001** |
| **Protein content (%)** | | |  |  |  |  |  |
| Linear term | 2.59 (4.04*10^-1^) | |  | **<0.001** | 1.76 (5.72*10^-1^) |  | **0.004** |
| Quadratic term | -3.67 (4.98*10^-1^) | |  | **<0.001** | -3.67 (6.93*10^-1^) |  | **<0.001** |
| Precipitation (mm) | -1.36*10^-2^ (9.44*10^-4^) | |  | **<0.001** | -1.02*10^-2^ (1.34*10^-3^) |  | **<0.001** |
| Whole-model (df = 32) | | | 0.88 | **<0.001** |  | 0.64 | **<0.001** |
| **Harvest Index (kg/kg)** | | |  |  |  |  |  |
| Linear term | -2.92*10^-1^ (9.79*10^-3^) | |  | **<0.001** | -3.71*10^-1^ (9.04*10^-3)^ |  | **<0.001** |
| Quadratic term | 5.30*10^-2^ (1.21*10^-2^) | |  | **<0.001** | 9.83*10^-2^ (1.10*10^-2^) |  | **<0.001** |
| Precipitation (mm) | 3.98*10^-4^ (2.29*10^-5^) | |  | **<0.001** | 3.83*10^-4^ (2.12*10^-5^) |  | **<0.001** |
| Whole-model (df = 32) | | | 0.97 | **<0.001** |  | 0.98 | **<0.001** |

**Table S6.** Levene’s test results for various traits in oat and barley over the summer generations. Statistical significance indicates that there exists a significant difference in within-trait variation between the groups compared.

|  | Oat | | | Barley | | |
| --- | --- | --- | --- | --- | --- | --- |
|  | df | F-value | P-value | df | F-value | P-value |
| Grain yield | 3; 32 | 2.488 | 0.08 | 3; 32 | 2.496 | 0.08 |
| Protein yield | 3; 32 | 1.864 | 0.16 | 3; 32 | 5.134 | **0.005** |
| Plant height | 3; 32 | 1.726 | 0**.**18 | 3; 32 | 5.769 | **0.002** |
| Root:Shoot ratio | 3; 32 | 1.696 | 0.19 | 3; 32 | 3.485 | **0.027** |
| Root length ratio | 3; 31 | 1.241 | 0.31 | 3; 32 | 4.131 | **0.014** |
| Grain yield:Control grain yield | 3; 32 | 3.513 | **0.026** | 3; 32 | 2.814 | 0.05 |

**Fig S1.** A climograph showing the average temperature (line) and total precipitation (bars) during the period form sowing until maturity in each field season of the group selection experiment (from 2018 to 2021).

**Fig S2.**  **Changes in crop root growth and biomass allocation for winter generations across eight generations of group selection.** Variation across group selected oat and barley are shown for the following traits: A-B, Root mass; C-D, Root dry mass to shoot dry mass ratio; E-F, Shoot elongation index (straw height to shoot dry mass ratio); G-H, Shoot dry mass. Shaded areas denote confidence 95% confidence intervals of the regression lines. Whole-model R^2^ and *P*-values are shown. For significance estimates for quadratic and linear terms, see Table S2. Note that the experiment comprised winter (1, 2, 5, 7) and summer (2, 4, 6, 8) generations; only the winter generations, which experienced greenhouse conditions are shown.
